# Progress with accreditation and standards in the Middle East: the cases of the Jordanian Health Care Accreditation Council and Egyptian General Authority for Healthcare Accreditation and Regulation

**DOI:** 10.1093/intqhc/mzag076

**Published:** 2026-05-29

**Authors:** Jeffrey Braithwaite, Salma Joauni, Ahmed A Taha, Rehab Mahmoud, Rabab Diab, Thaira Madi, Toqa Jamhawi, Omaima Nassar, Worood Al Khub, Rania Medhat, Doaa Elsherif, Carsten Engel, Ellen Joan van Vliet, Ezequiel Garcia-Elorrio

**Affiliations:** Australian Institute of Health Innovation, 75 Talavera Road, Macquarie University, Sydney, NSW, 2109, Australia; Health Care Accreditation Council (HCAC), Al Raafah Complex, Abdallah Ghosheh St. 58, Amman, Jordan; General Authority for Healthcare Accreditation and Regulation (GAHAR), Cairo, Kafr ash Shara'inah، الظهير الصحراوى لمحافظة القاهرة،،, Cairo Governorate Desert, Cairo Governorate, 4851415, Egypt; General Authority for Healthcare Accreditation and Regulation (GAHAR), Cairo, Kafr ash Shara'inah، الظهير الصحراوى لمحافظة القاهرة،،, Cairo Governorate Desert, Cairo Governorate, 4851415, Egypt; Health Care Accreditation Council (HCAC), Al Raafah Complex, Abdallah Ghosheh St. 58, Amman, Jordan; Health Care Accreditation Council (HCAC), Al Raafah Complex, Abdallah Ghosheh St. 58, Amman, Jordan; Health Care Accreditation Council (HCAC), Al Raafah Complex, Abdallah Ghosheh St. 58, Amman, Jordan; Health Care Accreditation Council (HCAC), Al Raafah Complex, Abdallah Ghosheh St. 58, Amman, Jordan; Health Care Accreditation Council (HCAC), Al Raafah Complex, Abdallah Ghosheh St. 58, Amman, Jordan; General Authority for Healthcare Accreditation and Regulation (GAHAR), Cairo, Kafr ash Shara'inah، الظهير الصحراوى لمحافظة القاهرة،،, Cairo Governorate Desert, Cairo Governorate, 4851415, Egypt; General Authority for Healthcare Accreditation and Regulation (GAHAR), Cairo, Kafr ash Shara'inah، الظهير الصحراوى لمحافظة القاهرة،،, Cairo Governorate Desert, Cairo Governorate, 4851415, Egypt; International Society for Quality in Health Care (ISQua), Dublin, Suite 210, 2nd Floor, South Point, Herbert House, Harmony Row, Dublin, D02 H270, Ireland; Qualicor Europe, Churchilllaan 11, Utrecht, 3527 GV, The Netherlands; Institute for Clinical Effectiveness and Health Policy, Dr. Emilio Ravignani 2024, Autónoma de Buenos Aires, C1414 Cdad., Argentina

**Keywords:** Accreditation, Standards, Quality of care, Patient safety, External evaluation organisations, International healthcare

## Abstract

Accreditation has become a central strategy for regulating and improving quality in health systems worldwide. Evidence suggests accreditation can strengthen governance, standardise processes and build quality improvement capability, although effects on outcomes remain variable and context-dependent. This paper examines two leading Middle Eastern accreditation bodies: the Jordanian Health Care Accreditation Council (HCAC) and Egypt’s General Authority for Healthcare Accreditation and Regulation (GAHAR). Both organisations are accredited by the International Society for Quality in Health Care External Evaluation Association (ISQua EEA), signalling alignment with international benchmarks while adapting standards to local contexts. We describe their origins, mandates, standards portfolios and system roles, and interpret their trajectories in light of recent global syntheses of the accreditation literature. We then outline HCAC’s and GAHAR’s key challenges and strategic opportunities over the next five years, including their evaluation and use of large language models, and the contributions they can make to patient and community engagement, equity, digital health, climate change, and organisational resilience. The experiences of HCAC and GAHAR offer lessons for similarly reforming health systems both in the Middle East, and other regions.

## Introduction

Accreditation has become a dominant quality and regulatory mechanism in healthcare worldwide. Three decades of research show it can strengthen governance, standardize clinical and organizational processes, enhance patient safety, and build improvement capability, though outcomes vary with context [1, 2]. A recent AI-enabled synthesis by Patel *et al.* from the Australian Institute of Health Innovation (AIHI) for the International Society for Quality in Health Care (ISQua) highlights global accreditation trends: system maturity, local context, and growing emphasis on patient engagement, equity and digital health [3].

Within this landscape, Jordan’s and Egypt’s health systems have rapidly evolved. Jordan’s Health Care Accreditation Council (HCAC) and Egypt’s General Authority for Healthcare Accreditation and Regulation (GAHAR) are national bodies for standards development and accreditation. Both have attained ISQua External Evaluation Association (ISQua EEA) recognition, signalling alignment with international best practice and positioning them as regional leaders [4–7]. HCAC and GAHAR illustrate the maturation of Middle East accreditation: from compliance-oriented programmes to nationally embedded governance mechanisms. The next phase of accreditation will depend on more effective evaluation, explicit attention to implementation burden and paper compliance, and closer alignment with person-centred, equitable and learning-oriented reforms.

## Jordan’s HCAC

Established in 2007 as a non-profit shareholding company, HCAC was mandated to elevate quality and patient safety through national standards and accreditation [4, 5]. It develops evidence-informed care standards, trains surveyors, supports accreditation preparation, and consults across public, private and non‑governmental sectors. A regional pioneer, HCAC was the first Eastern Mediterranean organization to receive all three ISQua EEA accreditations: for the organization, its standards, and its surveyor training programme, demonstrating rigorous international alignment [6, 7].

HCAC has progressively expanded its portfolio. Beginning with hospital standards, it introduced accreditation for primary healthcare, family planning, laboratories, diabetes and cardiovascular programmes, medical transport, and, uniquely, for mammography units. It developed standards for ambulatory services, including dental, radiology, physiotherapy, ophthalmology, and dermatology, and for disability services, shelters, and services for women and children in need [5, 6]. This reflects a system-level accreditation approach to improve quality across the health and social care continuum.

HCAC complements standards with quality improvement initiatives, including National Quality and Safety Goals, a “Change Day,” a national quality competition, and a biennial international quality conference. Its ISQua-accredited surveyor programme professionalises surveying by involving experts from policy, clinical and management backgrounds [5–7]. These initiatives underscore how HCAC has moved beyond episodic assessment towards a broader culture of continuous quality improvement.

## Egypt’s GAHAR

Founded in 2018 under Egypt’s Universal Health Insurance Law, GAHAR functions as an autonomous body responsible for developing, issuing and updating national healthcare accreditation and regulatory standards [8]. It sets standards for public and private sectors, accredits facilities in the universal insurance scheme, and supports continuous improvement in patient safety and quality.

GAHAR has an integrated suite of national accreditation standards, aligned with international best practices, adapted to the Egyptian healthcare context. These span hospitals, primary healthcare, mental health, long-term care and rehabilitation, ambulatory and outpatient services, laboratories, imaging, physiotherapy, wellness, and specialized care [8, 9]. These standards emphasize governance, patient safety, accessibility, integrated services, infection prevention and control, information management, workforce development, environmental safety, risk management and patient‑centredness, drawing on WHO and ISQua principles [3, 8, 9].

GAHAR also incorporates Green and Sustainable Healthcare Facilities Requirements into accreditation and has issued a National Guideline for Medical Equipment Management, strengthening technology governance across equipment lifecycles. Beyond accreditation, GAHAR provides technical guidance, training and regulatory oversight to ensure ongoing quality and safety [8, 9]. GAHAR thus functions not only as an accreditor but as a reform instrument within Egypt’s wider health-system transformation.

## Accreditation in practice

Both organizations face rising demand, constrained resources and heightened expectations for quality and transparency. HCAC’s expanding coverage shows how accreditation can extend across health and social sectors [5, 6]. GAHAR’s remit spans diverse facility types within universal health insurance reforms, making accreditation a key mechanism linking quality to national financing [8, 9]. This distinctiveness is clear: HCAC is notable for breadth across service lines and sector-wide quality initiatives, whereas GAHAR is notable for formally integrating financing reform and national regulatory oversight. This contrast offers models from which other national accreditation and quality improvement bodies can learn.

Patel et al. identify characteristics of high‑performing accreditation systems: evidence‑based standards, contextual sensitivity, capability building and a shift from compliance to continuous improvement [3]. HCAC and GAHAR increasingly display these attributes through different pathways [8–12].

The literature identifies areas where accreditation’s impact is difficult to attribute or sustain, including to improve clinical outcomes, cost and equity [1–3]. This reflects the familiar attribution problem: in complex health systems, outcomes cannot be ascribed to accreditation alone. Nor should accreditation be treated as a stand-alone causal lever. And poorly aligned standards, or undue focus on survey performance, can generate administrative burden, staff fatigue and ritualized compliance rather than deeper improvement. These limitations matter as Middle Eastern systems transition from adoption to consolidation.

## Looking ahead: 2026–2030

Looking to the next 5 years, both HCAC and GAHAR face challenges, yet possess many strengths (Table 1).

**Table 1:** Challenges and strengths encountered by HCAC and GAHAR in the next 5 years.

Category	Key area	Detailed explanation
Challenges	Resource constraints and implementation capacity	Facilities with limited infrastructure, staffing, leadership capability or effective health information systems may struggle to meet standards without targeted and concerted support. Where workforce capacity is already stretched, the implementation burden of accreditation can itself become a barrier to improvement.
	Balancing rigour and feasibility	Accreditation bodies must calibrate standards to occupy the sweet spot, being both aspirational and achievable, especially where baseline quality varies widely.
	Avoiding ‘paper compliance’	The risk that documentation and ‘inspection readiness’ overshadow deeper cultural and behavioural change remains real. Without attention to staff workload, local context and organizational culture, accreditation can become a documentation exercise that consumes improvement time rather than strengthening practice.
	Measuring impact	Robust evaluation of accreditation’s effects on safety, quality, workforce well-being and patient experience is still developing in the region.
	Sustaining post- accreditation improvements	Maintaining outcomes requires a quality-focused culture, continuous monitoring and leadership support.
Strengths	A store of expertise	Low- and middle-income settings have often lacked the resources to develop quality initiatives and strategies when basic care and universal coverage are key priorities. HCAC and GAHAR have carefully and persistently developed core knowledge and skills and shared these widely.
	National governance	HCAC and GAHAR have been key vectors for a focus on quality and safety in their respective systems [8–12]. Without that leadership positioning, there would doubtless be a leadership vacuum in quality and safety; this would need to be addressed if care quality is to be emphasized.
	Regional leadership	HCAC in particular has emerged as a reference model for neighbouring countries, offering expertise in standards development, surveyor training and quality improvement programmes [8–12].
	Reform alignment	GAHAR’s position within Egypt’s universal health insurance reform creates a powerful lever: accreditation becomes a gateway to participation in national financing, fostering strong incentives for providers to improve [4–6].

HCAC and GAHAR are positioned to consolidate their accreditation programmes and influence broader safety and quality agendas. Five priority directions emerge from Patel *et al.*’s synthesis and wider literature [1–3, 12, 13]: Deepening the evidence base and evaluation; embedding patient, family, and community voices and expertise; addressing equity and variation; harnessing digital health and AI responsibly; and supporting resilient, learning organizations.

All-in-all, HCAC and GAHAR are showing how accreditation can strengthen safety, quality and governance in Middle Eastern health systems. Both organizations now stand at an inflection point: moving from first-generation compliance models toward second-generation accreditation focused on learning, equity, person centred-care, digital readiness and resilience [1–3]. The next phase should not focus on excessive accreditation documentation, but whether accreditation helps organizations improve real-world care, reduces unwarranted variation, and builds trust with patients, staff and communities.

## Data Availability

No new data were generated or analysed in support of this review. No primary data were collected. All data sources are publicly available and the website has been provided.
